# The Deaths of 94 Mental Health-care Users in Gauteng, South Africa

**DOI:** 10.3389/fpubh.2017.00126

**Published:** 2017-05-31

**Authors:** Catherine Govender

**Affiliations:** ^1^Psychology, University of South Africa, Pretoria, South Africa

**Keywords:** Esidimeni life, mental health-care users, Gauteng Department of Health, health ombudsman, Mental Health Review Board

South Africa has made strides toward creating policy and legislation to counteract the divisive rhetoric of apartheid-era mental health care (1). The Mental Healthcare Act of 2002 (2) repealed the individual mental health acts of the previous Bantustans and the Mental Health Act of 1973 to replace these with provisions for all South Africans with mental illnesses to be treated in a manner conducive to health and to be protected where necessary. The legislators encouraged a move toward community-based care in contrast to the centralized mandate of the apartheid government and provision was made for oversight in the form of Mental Health Review Boards (1, 2).

In recent months, the practical benefits yielded from such legislative changes were questioned following the deaths of 94 mental health-care users (MHCUs) in South Africa’s most densely populated province, Gauteng. The Gauteng Department of Health had embarked on a cost-cutting project, The Gauteng Mental Health Marathon Project, which saw the cancelation of a contract with the mental health-care facility, Life Esidimeni in 2016. As a result, 1,371 MHCUs were transferred from Esidimeni to multiple NGOs throughout the province (3). In September 2016, the public became aware of the deaths of 36 MHCUs through questions posed in parliament (4). In response, South Africa’s Minister of Health, Dr. Aaron Motsoaledi, appointed the Health Ombudsman to investigate these deaths (4).

Health Ombudsman, Professor Malegapuru Makgoba’s final report was released in February 2017. While this manuscript cannot convey the full complexity of the tragedy, some of the findings are commented on here. The report indicated that the MHCU transfer was carried out with increasing rapidity over 1 April to 30 June 2016. The Ombudsman provisionally attributed 94 deaths to the project and found that these patients died under “unlawful circumstances” (3) as they were transferred to various facilities that were not licensed to offer them the requisite care. The transfers were described as rushed, and deaths were attributed to starvation, dehydration, and possible infections resulting from lapses in infection control (3).

One aspect of the Ombudsman’s report that highlights South Africa’s shortfalls in transforming mental health care is the evidence on the lack of proper recording of data: patient records were incomplete or missing, and the paper trails were not backed up in any manner. The results were that patient treatment could not be verified, some families were not properly informed while others had no inkling that their kin had died or even that they had been moved. The provincial health department could not identify seven deceased who were later shown to be part of the cohort; did not know who died where; and did not have some of the deaths recorded (3). While integrated health systems are driving forces for improved health care in many countries, the developing world is still lagging behind in the use of health systems to improve practice through research (5). There have been efforts by institutions such as the Centre for Public Mental Health and works by *inter alia* Janse van Rensburg, Peterson, and Lund (1, 6, 7) to highlight the need for mental health data. In spite of these efforts, mental illness monitoring was conspicuously absent in the draft integrated national strategy for health research circulated for comment in 2016 (8). Once again, we are facing a marginalization of mental health care as commented on by Skeen and colleagues (6).

One other striking point made by the Ombudsman’s report is about the failure of the mental health review boards to carry out their mandate, which was to protect the most vulnerable from being moved (1). The review boards should have questioned the fitness of the receiving institutions to cater for the needs of the MHCUs and should have determined if the MHCUs were indeed in a state to be transferred at all. Instead, none of the 27 receiving NGOs were fit for the purpose of care but had been licensed by provincial officials to do so (3). It also does not seem that attention was paid to the provision of care in the period between cancelation of the Esidimeni contract and transferring the patients: some patients were reportedly received at the NGOs already in some state of neglect (3).

Perhaps there is one aspect of the Marathon Project that was correctly grasped by the mental health officials involved: mental health-care transformation is indeed a long and strategic run. Ironically, the same public servants treated it as a sprint and families are left mourning 94 of our own… and counting.

## Author Contributions

The author confirms being the sole contributor of this work and approved it for publication.

## Conflict of Interest Statement

The author declares that the research was conducted in the absence of any commercial or financial relationships that could be construed as a potential conflict of interest.
